# Metals strengthen with increasing temperature at extreme strain rates

**DOI:** 10.1038/s41586-024-07420-1

**Published:** 2024-05-22

**Authors:** Ian Dowding, Christopher A. Schuh

**Affiliations:** 1 Department of Materials Science and Engineering, Massachusetts Institute of Technology, Cambridge, MA USA; 2 Department of Materials Science and Engineering, Northwestern University, Evanston, IL USA

**Keywords:** Mechanical properties, Metals and alloys

## Abstract

The strength of materials depends on the rate at which they are tested, as defects, for example dislocations, that move in response to applied strains have intrinsic kinetic limitations^1–4^. As the deformation strain rate increases, more strengthening mechanisms become active and increase the strength^4–7^. However, the regime in which this transition happens has been difficult to access with traditional micromechanical strength measurements. Here, with microballistic impact testing at strain rates greater than 10^6^ s^−1^, and without shock conflation, we show that the strength of copper increases by about 30% for a 157 °C increase in temperature, an effect also observed in pure titanium and gold. This effect is counterintuitive, as almost all materials soften when heated under normal conditions. This anomalous thermal strengthening across several pure metals is the result of a change in the controlling deformation mechanism from thermally activated strengthening to ballistic transport of dislocations, which experience drag through phonon interactions^1,8–10^. These results point to a pathway to better model and predict materials properties under various extreme strain rate conditions, from high-speed manufacturing operations^11^ to hypersonic transport^12^.

## Abstract (web summary)

Microballistic impact testing at strain rates greater than 10^6^ s^−1^ shows that pure metals, including copper, gold and titanium, become stronger with increasing temperature.

## Main

Metals usually soften at high temperatures, because the motion of defects that control strength—dislocations—is thermally activated: temperature helps these carriers of plasticity move across the crystal lattice and bypass any obstacles there^6,10,13,14^. The softening of metals with temperature is extremely broadly observed, being the norm over more than ten orders of magnitude in strain rates, spanning 10^−6^–10^4^ s^−1^. However, as strain rates increase beyond 10^4^ s^−1^, more deformation mechanisms can become active; these contribute to a marked increase in strength through dislocation-drag-controlled plasticity^2,4,15,16^. Understanding material properties at such high strain rates is critical for designing and engineering new materials for use in extreme conditions. Deformation rates greater than 10^4^ s^−1^ are commonly seen in hypervelocity meteorite impacts^12^, high-speed metal machining^11^, sandblasting and erosion^17,18^ and in metal additive manufacturing processes such as cold spray^19^. The drag mechanisms that prevail under high rates are interesting because they are not rate limited in the same manner as the thermally activated mechanisms described above; they are instead expected to be a mode of ballistic dislocation transport. It has been anticipated that, in this extreme range, metals indeed may not soften at high temperatures^4,16,20,21^, a behaviour that is counterintuitive and at odds with decades of studies in less extreme conditions.

Unfortunately, experimental data on extreme strain-rate deformation mechanisms are rather rare, owing to limitations of measurement tools that can access extreme strain rates. Kolsky bars^22^, flyer plates^23^, shock impacts^24^, high-power laser pulses^25^, shockless isentropic compression experiments^26^ and gas-gun-driven projectiles^27^ are examples of methods that are generally conducted on macroscale samples (ranging from a few millimetres to a few hundred millimetres) and require high impact velocities (hundreds of metres per second) to induce these large strain rates. In general, the quantitative measurements provided by these methods end around a strain rate of about 10^4^ s^−1^. At higher rates, these approaches are more typically aimed at studying material spall^7^ and convolute the mechanical response with strong shock effects in the material during impact. We are not aware of data that are able to separate out shock effects and show the posited loss of thermally activated softening at high strain rates.

Advances in optically driven microballistic systems over the past decade have made it possible to accelerate 1–50-µm-sized impactors to velocities up to and beyond 1,000 m s^−1^, producing extreme strain rates from 10^6^ to 10^9^ s^−1^, strain rates on the same order of magnitude as, for example, discrete dislocation and molecular dynamics simulations^28,29^. Notably, because of the small scale of the test, these rates are achieved without necessarily passing into the strong shock regime that characterizes many other rapid experimental test methods, permitting a clean assessment of material strength under extreme rates. There are now at least two different means of using such tests to extract quantitative measures of strength from an impacted substrate: dynamic yield strengths can be extracted from the rebound behaviour of the impactor over a range of velocities^30–32^ and dynamic hardness values can be measured on the basis of post-impact measurements of the impact crater sizes^33^. The availability of these strength measures at extreme rates opens the door to exploration of the drag mechanisms that are expected to dominate there, but are quantitatively underexplored.

## Quantitative measurements of strength and hardness

**Fig. 1:**
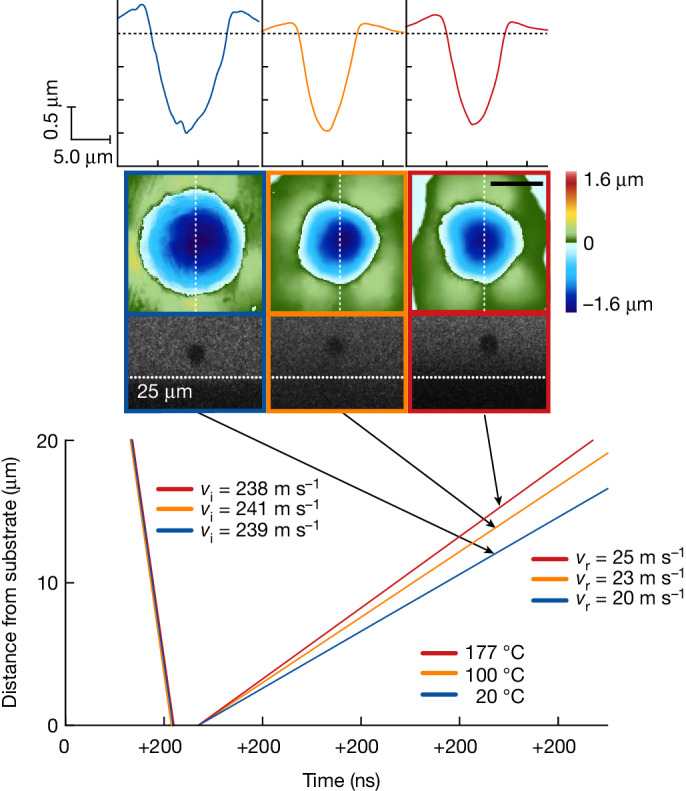
Impact trajectories and impact craters. The impact and rebound trajectories of three alumina particles impacting a copper substrate at 20 °C (blue), 100 °C (orange) and 177 °C (red). As the temperature is increased, the rebound velocity of the impacting particle increases, as shown by both the plotted rebound trajectories as well as the grey insets of the particle 600 ns after rebound. Insets of plan and line-scan views of each impact crater, measured with 3D laser scanning microscopy, show that, as the temperature is increased, the impact crater depth and width decrease. Scale bar, 5 μm.

High-strain-rate micromechanical impact experiments were carried out using laser-induced particle impact tests, detailed in Methods. Spherical alumina microparticles, roughly 12.5 ± 1 μm in size, were chosen as impactors onto copper substrates owing to their much higher hardness (static hardness 15.7 versus 0.56 GPa for annealed Cu)^34,35^, to isolate the plasticity to the substrate. Figure 1 shows some typical results, in which we track by high-speed camera the inbound and rebound trajectories of alumina particles as they impact the copper substrate. The three examples in Fig. 1 are chosen for the close similarity of the conditions: these three nominally identical particles are launched at almost the same velocity and thus follow the same inbound trajectories very closely. However, these three experiments were conducted at 20, 100 and 177 °C and, as the test temperature is increased, the particle rebounds more aggressively, as shown by the increasing rebound trajectories, as well as the differences in height in the inset black and white photographs of the particles 600 ns after impact. These results indicate a higher strength of copper at higher temperatures, as more kinetic energy is carried away in the rebound; this is a first signature of anomalous temperature dependence of strength at high rates.

The raw trajectory data in Fig. 1 are corroborated by an independent measurement of local plastic deformation. The upper insets in Fig. 1 show impact crater profile measurements using 3D laser scanning confocal microscopy for the same three impacts, in profile (top row) and in plan-view projection (middle row); the crater diameter and depth decrease with test temperature at the same impact velocity. Quantitatively, the crater volume decreases by about a factor of two between room temperature and 177 °C. Because crater volume is inversely proportional to strength^36^, the crater images in Fig. 1 independently confirm that copper is hardening at higher temperatures under these extreme impact conditions.

**Fig. 2:**
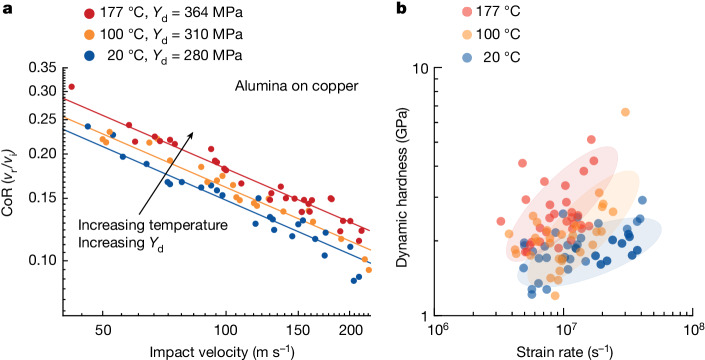
Copper dynamic strength and hardness. **a**, The impact velocity versus CoR, *v*_r_/*v*_i_, is plotted on a double-logarithmic scale for a range of impact velocities. Across the range of impact velocities, the impacts at higher temperatures led to a faster rebound. The solid lines represent the scaling law for ideally plastic impacts with the fitting parameter, *Y*_d_, increasing with temperature. Uncertainty in measured velocities is ±2%, which is on the order of the size of the data markers. **b**, Dynamic hardness measurements from each impact are plotted against deformation strain rate. The shaded regions serve as a guide for the eye to show where most of the hardness values fall for each temperature. As with strength, the hardness trends upward with increasing temperature and measurement uncertainties are on the order of the size of the data markers.

The two independent signatures of thermal hardening in Fig. 1—the increased rebound and the decreased crater volume—are both amenable to quantitative analysis to extract strength measures and, in what follows, we take each of these in turn. We first look at a larger set of rebound data for many more impact experiments. The rebound response is quantified using the coefficient of restitution, CoR = *v*_r_/*v*_i_, the ratio of the rebound and impact velocities. Figure 2a plots the CoR as a function of the impact velocity in double-logarithmic format. For a given test temperature, higher impact velocities lead to lesser rebounds, as more energy is dissipated in plasticity of the substrate. In fact, the parabolic scaling in Fig. 2a is the expected form for plasticity, as developed by Wu et al.^30,31^, who showed that ideally plastic impacts follow the power-law relation:

1 $${\rm{C}}{\rm{o}}{\rm{R}}=\frac{{v}_{{\rm{r}}}}{{v}_{{\rm{i}}}}=\alpha {\left(\frac{{v}_{{\rm{y}}}}{{v}_{{\rm{i}}}}\times \frac{{E}^{\ast }}{{Y}_{{\rm{d}}}}\right)}^{1/2}$$

in which the coefficient *α* = 0.78 for an undeforming impactor on a deforming substrate, *Y*_d_ is the dynamic yield strength of the substrate, E* is an effective elastic modulus of the materials involved and *v*_y_ is a characteristic velocity at which plastic deformation is initiated (see Supplementary Information Section 1 for more details). Not only do the data in Fig. 2a conform to the scaling of equation (1) but the equation is fully predictive with the fitting of a single parameter, namely the dynamic yield strength *Y*_d_, which is an average strength measure describing the complex deformation of the substrate over the entire duration of an impact. At room temperature, fitting the CoR data in Fig. 2a yields a dynamic strength of 280 MPa for copper at the average strain rate of 10^7^ s^−1^ for these tests. For comparison, under quasistatic conditions, annealed copper would have a typical strength on the order of 70 MPa (ref. ^37^) and under extreme conditions involving shock (such as Richtmyer–Meshkov instability tests^38^), strengths up to 1,000 MPa have been inferred. The present value between these limits represents substantial rate-hardening, but no superimposed effect of shock or high pressure. One advantage of this assessment of strength is that it averages out local heterogeneities in the microstructure (including grain boundaries, crystal orientation effects and defect structures) over the entire range of impact velocities; these effects contribute to the scatter in the data and provide a quantitative measurement uncertainty as a standard deviation of the data’s departure from the trendline in Fig. 2a.

The more interesting trend in Fig. 2a is the effect of the test temperature on the CoR curves and the dynamic yield strengths they encapsulate. As seen previously in Fig. 1 for several example cases, we now see very broadly that temperature promotes greater rebounds in copper, over the full range of tested velocities. The fitted solid lines reflect an increasing dynamic strength: *Y*_d,20°C_ = 280 MPa, *Y*_d,100°C_ = 310 MPa and *Y*_d,177°C_ = 364 MPa. With increasing temperature, the dynamic yield strength of copper increases by roughly 30% over a range of about 150 °C.

One limitation of the CoR analysis above is that it averages the dynamic strength over a range of velocities because it requires the fitting of many data points together. A second strength measure, the dynamic hardness, permits a test-by-test analysis of the trends shown above. Dynamic hardness measurements also require the quantitative measurement of inbound and outbound velocities to assess the amount of energy deposited into the substrate and expended as plastic deformation, normalized by the crater volume, *V* (ref. ^33^):

2 $${H}_{{\rm{d}}}=\frac{1/2\times {m}_{{\rm{p}}}\times ({v}_{{\rm{i}}}^{2}-{v}_{{\rm{r}}}^{2})}{V}$$

in which *m*_p_ is the mass of the particle. The crater volume can be accurately assessed with scanning confocal microscopy as in Fig. 1 and, combined with the associated velocity measurements, gives the dynamic hardness of copper for each individual experiment. The results of this analysis are plotted in Fig. 2b for the range of strain rates tested, with the net strain rate of a given experiment assigned as the ratio of the impact velocity to the particle diameter, *v*_i_/*d*. Clearly, beyond the intrinsic scatter of the experiments, these data confirm all of the observations above: at strain rates greater than about 10^6^ s^−1^, the hardness of copper increases with increasing test temperature.

**Extended Data Fig. 1:**
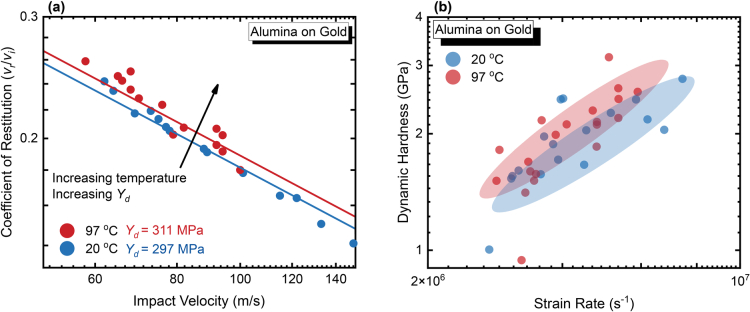
Gold dynamic strength and hardness. **a**, The impact velocity versus CoR is plotted on a double-logarithmic scale for alumina impacts on gold substrates at 20 °C (blue) and 97 °C (red). Using scaling for ideally plastic impacts, the dynamic yield strength for each impact temperature is calculated to be *Y*_d,20°C_ = 297 MPa and *Y*_d,97°C_ = 311 MPa. Uncertainty in measured velocities is ±2%, which is on the order of the size of the data markers. **b**, The dynamic hardness of titanium is plotted for both 20 °C (blue) and 97 °C (red). For impact at similar strain rates, the tests at 97 °C show a higher hardness, and measurement uncertainties are on the order of the size of the data markers.

**Extended Data Fig. 2:**
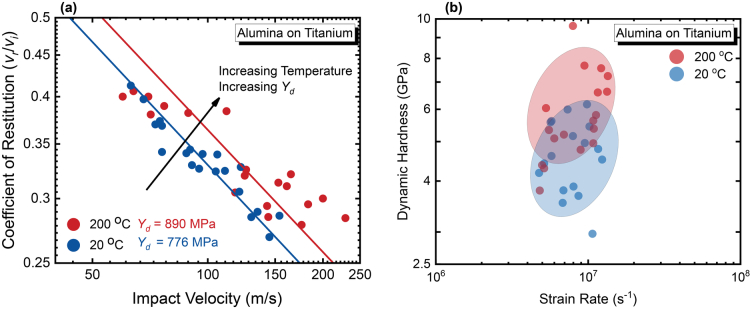
Titanium dynamic strength and hardness. **a**, The impact velocity versus CoR is plotted on a double-logarithmic scale for alumina impacts on titanium substrates at 20 °C (blue) and 200 °C (red). *Y*_d_ for each impact temperature is calculated to be *Y*_d,20°C_ = 776 MPa and *Y*_d,200°C_ = 890 MPa. Uncertainty in measured velocities is ±2%, which is on the order of the size of the data markers. **b**, *H*_d_ of titanium is plotted for both 20 °C (blue) and 200 °C (red). For impact at similar strain rates, the tests at 200 °C show a higher hardness, and measurement uncertainties are on the order of the size of the data markers.

The above results represent the first experimental data to show anomalous temperature dependence at strain rates greater than 10^6^ s^−1^, in experiments that are both quantitative and free of strong shock effects. This result is also not limited to pure copper; we have observed the same trends in pure Au and Ti as well, as shown in Extended Data Figs. 1 and 2. For all three metals, the two independent strength measures (dynamic yield strength and dynamic hardness) reflect a change in deformation mechanism owing to the synergy between strain rate and temperature; at extreme strain rates, all three metals harden when heated.

## Deformation mechanisms

**Fig. 3:**
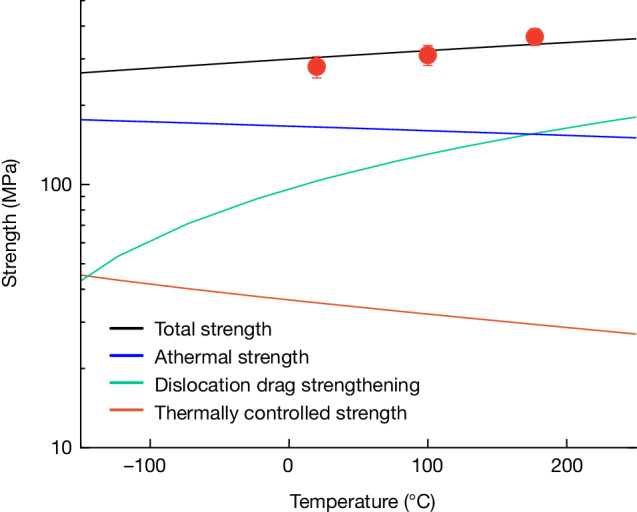
Contributions of each strengthening mechanism. The contribution of each strength term for pure copper, calculated for a range of temperatures at a fixed strain rate of 10^7^ s^−1^. The magnitude of the increase in dislocation drag strengthening is greater than the magnitude of the decrease in all other strength terms, leading to a net increase in the total strength with temperature at this extreme strain rate. The experimental data points, plotted in red, are in good agreement with the total strength resulting from the summation of each strength component. Error bars on the experimental data points are the standard deviation of the strength measurements.

The flow strength in pure metals can be broken down into three additive components: thermal, athermal and drag strengthening^5,39,40^, each of which is plotted separately in Fig. 3 at our experimental strain rate of 10^7^ s^−1^:

- The thermal strength component comes from dislocation interactions with short-range barriers (Peierls–Nabarro barriers, vacancies) that can be fully overcome by thermal fluctuations. This strength can be calculated as a function of temperature and strain rate following refs. ^8,41,42^ as described in Supplementary Information Section 2.1 and follows a conventional thermally activated softening-with-temperature trend (red line in Fig. 3).
- The athermal strength component arises from other barriers to dislocation motion that are not able to be overcome by thermal fluctuations alone, such as dislocation–grain boundary and dislocation–forest interactions^8^. As shown in Supplementary Information Section 2.2, the total athermal strength component of copper can be calculated with standard models for our strain rate of 10^7^ s^−1^, giving the blue line in Fig. 3, again a softening-with-temperature trend. The softening in this case is because of the lowering of the elastic modulus with temperature, which—in turn—lowers the energy of dislocation–obstacle interactions.
- The final strength component, drag strengthening, comes from the interaction of dislocations with phonons in the lattice^43^. This interaction gives rise to anomalous thermal strengthening, because higher temperatures increase the energy levels of phonons and thus increase the interaction of dislocations with them. Supplementary Information Section 2.3 presents the standard model for phonon drag, which, when evaluated with inputs for copper at our strain rate of 10^7^ s^−1^, gives the green line in Fig. 3.

Although the first two contributions exhibit classical thermal softening, the third term has a large influence under high-rate conditions, which explains why the dynamic strength and hardness increase at our experimental strain rates. In fact, the increase predicted by the summation of the three terms are reasonably aligned in both absolute magnitude and the scale of the change with temperature, as shown by the black line in Fig. 3.

**Fig. 4:**
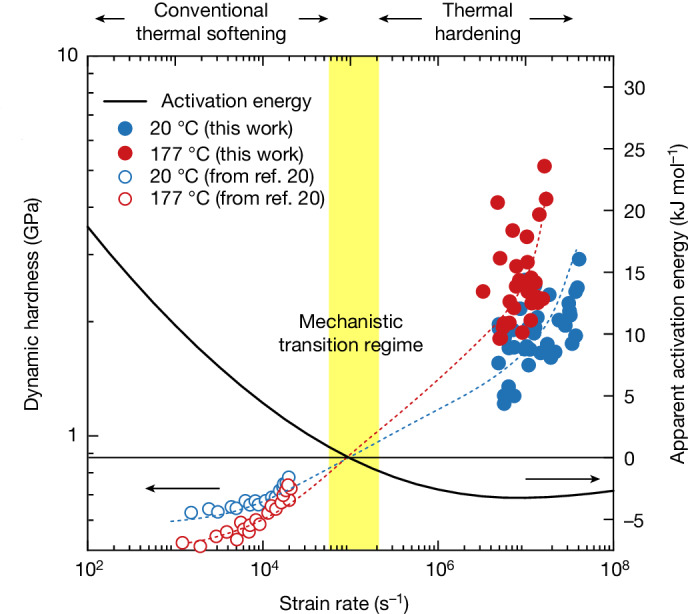
Apparent activation energy for plasticity. The blue and red dashed lines serve as guides for the eye to connect hardness values, converted from strength values from ref. ^20^ (open circles), at low rates and dynamic hardness values from this work (filled circles). The apparent activation energy, *Q*_app_, for plasticity (solid black line) of copper is also plotted as a function of strain rate at a constant temperature of 20 °C based on the model in the Supplementary Information. At rates below roughly 10^5^ s^−1^, *Q*_app_ changes from positive, indicating thermally activated dislocation motion, to negative, one of the necessary conditions for phonon drag to be substantial.

The unusual hotter-is-stronger trend in the present data are strictly an extreme-rate effect and, in fact, the experimental data in Fig. 2b allude to a crossover point at which the temperature dependence of strength inflects; at low enough rates, the data suggest a transition to classical thermally activated behaviour. This is elaborated more clearly in Fig. 4, in which we reproduce data from Fig. 2b, and now include literature data for copper of similar purity^20^, tested at the closest lower strain rates at which data are available (approximately 2 × 10^4^ s^−1^). The data at those lower rates exhibit the opposite temperature dependence from the present data. A clear transition regime exists between strain rates at which those literature data end and our data begin, as seen in the crossover of the blue and red dotted schematic trendlines. The highlighted yellow band shows the range of strain rates at which the crossover must occur. The strength equations described above can be used to evaluate the putative position of this crossover as well, by assessing the apparent activation energy for plasticity, *Q*_app_:

3 $${Q}_{{\rm{app}}}=R{\left(\frac{\partial {\rm{ln}}\dot{\varepsilon }}{\partial {\rm{ln}}\sigma }\right)}_{T}{\left(\frac{\partial {\rm{ln}}\sigma }{\partial (1/T)}\right)}_{\dot{\varepsilon }}$$

in which *R* is the gas constant. A theoretical value for the apparent activation energy for deformation can be determined from the strength equations in Supplementary Information Section 2 through the use of equation (3) and signals the tendency of the temperature dependence: positive values of *Q*_app_ suggest thermal softening owing to thermal hopping of dislocations, whereas negative values are required to capture thermal hardening, as in our experiments, by virtue of a transition into a ballistic-like transport regime of dislocation motion^1,44^ (more details are provided in Supplementary Information Section 3). The theoretical prediction for *Q*_app_ is plotted for copper at a constant temperature of 20 °C on the secondary *y* axis in Fig. 4. Indeed, the model suggests a critical strain rate near 10^5^ s^−1^, at which *Q*_app_ changes sign from positive to negative, signalling the change in controlling mechanism from thermally activated softening to dislocation drag strengthening in the ballistic regime. This result provides interesting new insight on previous work such as that in refs. ^45,46^, in which thermal softening was observed in copper even at rates as high as about 7 × 10^4^ s^−1^; the present results clarify that only at even higher rates above 10^5^ s^−1^ is the crossover expected.

## Implications and conclusions

Although models such as those used here have been in the literature for many years, microballistic testing provides a new opportunity to quantitatively measure the strength and hardness of materials at extreme strain rates, without complicating factors caused by strong shock physics. This not only provides a chance to test those models, as done here, but potentially to rapidly calibrate them for new materials and situations. The present data for three pure metals provides a starting point for focused study on those difficult-to-access mechanisms. The result that pure metals show marked thermal hardening at high strain rates also alludes to new strategies for materials design for extreme conditions; extrapolating conventional strength measurements to extreme conditions may lead not only to incorrect strength expectations but even incorrect directional dependencies of those strengths. For example, although pure copper is a soft metal at low strain rates and would normally be expected to soften at high temperatures, the strength of copper measured here at 10^7^ s^−1^ and 177 °C is more than 300 MPa; this is comparable with the conventional strength of 304 steel at this temperature^43^. The present results thus bolster the call for new modes of thinking about materials optimization for extreme conditions^47,48^.

## Methods

Spherical alumina powder particles, with nominal particle size of approximately 12.5 ± 1 μm, were purchased from Inframat Advanced Materials and used for microparticle impact experiments on copper and titanium, and nominal particle size of about 20 ± 1 μm for impact experiments on gold. Oxygen-free high-conductivity copper plates (OnlineMetals) and pure Ti rods (Beantown Chemical) were cut using a diamond saw and annealed at 600 °C for 10 h. Pure gold rods (Beantown Chemical) were cold rolled to an 85% reduction in thickness before being annealed at 125 °C for 3 h. The plates and rods were then polished using standard metallographic techniques to a 0.04-μm mirror finish.

We performed microballistic impacts using a laser-induced particle impact test, detailed elsewhere^28^. Launching pads for use at elevated temperatures consisted of a 210-μm glass slide with a 90-nm chromium layer sputter coated on top, a UV-curable adhesive and a second 210-μm glass slide, a construction specifically designed to attain high test temperatures without any materials degradation^28^. A monolayer of alumina particles was finely dispersed on top of the second glass slide using a drop of ethanol. A single particle was selected for impact based on predetermined parameters: size, shape and surface morphology. A laser pulse (Nd:YAG, 532 nm wavelength) was focused directly behind the particle, resulting in the ablation of the chromium layer and rapid acceleration of the particle towards the copper substrate placed 750 μm away. The velocity of the particle was predetermined by changing the laser pulse energy between 1 and 80 mJ. The velocities targeted in this study (below 250 m s^−1^ for copper and titanium and below 150 m s^−1^ for gold) are all slightly low compared with conventional high-rate testing; this is far below the point at which instabilities associated with hydrodynamic jetting/spall set on (approximately 650 m s^−1^ for the present geometry in ref. ^32^) and even farther from the regime of hydrodynamic penetration (about 900 m s^−1^ from ref. ^49^). Every impact falls within a regime in which no fracture-like events occur that could lead to fragmentation and ejection of the substrate material. The onset of such events would be identifiable by an excess loss of rebound velocity, seen as deviations from the power law in Fig. 2a (ref. ^32^), as well as post-mortem analysis of the craters^50^; nothing seen in our data suggests any material fragmentation or ejection, as expected for these conditions. Furthermore, all of the impact velocities for each material are also well above the elastic–plastic transition velocity (*v*_y_ from equation (S2) in the Supplementary Information). Plastic deformation sets on at velocities below 1 m s^−1^ for each material in this study, meaning that each impact in this work leaves a plastic crater in the substrate. Our procedure includes particle-wise measurements of impact velocity and particle diameter, thus providing a net strain rate for every individual experiment as *v*_i_/*d* (ref. ^33^); this quantity can be regarded as an average over the plastically deformed region over the duration of the impact event.

The flight, impact and rebound of the particle were captured using a high-speed camera (Specialised Imaging) able to capture 16 images with as small as 5 ns exposure times, with the event being illuminated by a second laser pulse (640 nm wavelength, 10 μs duration). The impact and rebound velocities of the particle were measured between frames. To conduct impacts at elevated temperatures, a custom resistive heating stage was fabricated using a machinable ceramic (Macor, McMaster-Carr) and nichrome wire (McMaster-Carr). Three thermocouples were placed around the substrate to ensure that a homogenous temperature distribution was reached. Before launch, the particles are heated by virtue of their proximity with the target substrate, with an offset distance of only 750 µm; the system was thermally equilibrated before launch, so particle, vapour and substrate are all at the same nominal temperature. Post-mortem analysis of the impact sites was conducted using 3D laser scanning confocal microscopy (VK-X200 Keyence) to measure the volume of the impact craters. Hardness measurements use this direct measurement of crater volume in equation (2).

## Online content

Any methods, additional references, Nature Portfolio reporting summaries, source data, extended data, supplementary information, acknowledgements, peer review information; details of author contributions and competing interests; and statements of data and code availability are available at 10.1038/s41586-024-07420-1.

### Supplementary information

Supplementary Information Supplementary Methods.

## Data Availability

All data are available in the main text or the Supplementary Information.

## References

[1] Kocks UF, Argon AS, Ashby MF (1975). Thermodynamics and kinetics of slip. Prog. Mater. Sci..

[2] Ashby MF (1972). A first report on deformation-mechanism maps. Acta Metall..

[3] Zerilli FJ, Armstrong RW (1987). Dislocation-mechanics-based constitutive relations for material dynamics calculations. J. Appl. Phys..

[4] Sargent, P. M. & Ashby, M. F. *The Presentation of High Strain-Rates on Deformation Mechanism Maps* ADA128822 (Defense Technical Information Center, 1983).

[5] Armstrong RW, Li Q (2015). Dislocation mechanics of high-rate deformations. Metall. Mater. Trans. A.

[6] Meyers, M. A. & Chawla, K. K. *Mechanical Metallurgy: Principles and Applications* (Prentice-Hall, 1984).

[7] Whelchel RL, Kennedy GB, Dwivedi SK, Sanders TH, Thadhani NN (2013). Spall behavior of rolled aluminum 5083-H116 plate. J. Appl. Phys..

[8] Liu R, Salahshoor M, Melkote SN, Marusich T (2015). A unified material model including dislocation drag and its application to simulation of orthogonal cutting of OFHC Copper. J. Mater. Process. Technol..

[9] Wulf GL (1979). High strain rate compression of titanium and some titanium alloys. Int. J. Mech. Sci..

[10] Armstrong RW, Walley SM (2008). High strain rate properties of metals and alloys. Int. Mater. Rev..

[11] Abukhshim NA, Mativenga PT, Sheikh MA (2006). Heat generation and temperature prediction in metal cutting: a review and implications for high speed machining. Int. J. Mach. Tools Manuf..

[12] Johnson BC, Minton DA, Melosh HJ, Zuber MT (2015). Impact jetting as the origin of chondrules. Nature.

[13] Armstrong RW, Arnold W, Zerilli FJ (2009). Dislocation mechanics of copper and iron in high rate deformation tests. J. Appl. Phys..

[14] Follansbee PS, Kocks UF (1988). A constitutive description of the deformation of copper based on the use of the mechanical threshold stress as an internal state variable. Acta Metall..

[15] Kumar A, Kumble RG (1969). Viscous drag on dislocations at high strain rates in copper. J. Appl. Phys..

[16] Kanel, G. I., Fortov, V. E. & Razorenov, S. V. *Shock-Wave Phenomena and the Properties of Condensed Matter* (Springer, 2004).

[17] Slikkerveer PJ, Beuten PCP, in’t Veld FH, Schollen H (1998). Erosion and damage by sharp particles. Wear.

[18] Hassani-Gangaraj M, Veysset D, Nelson KA, Schuh CA (2018). Melt-driven erosion in microparticle impact. Nat. Commun..

[19] Assadi H, Kreye H, Gärtner F, Klassen T (2016). Cold spraying – a materials perspective. Acta Mater..

[20] Sakino K (2000). Transition in rate controlling mechanism of FFC metals at very high strain rates and high temperatures. J. Phys. IV Fr..

[21] Tang Q, Hassani M (2024). Quantifying dislocation drag at high strain rates with laser-induced microprojectile impact. Int. J. Plast..

[22] Nicholas T (1981). Tensile testing of materials at high rates of strain. Exp. Mech..

[23] Curtis AD, Banishev AA, Shaw WL, Dlott DD (2014). Laser-driven flyer plates for shock compression science: launch and target impact probed by photon Doppler velocimetry. Rev. Sci. Instrum..

[24] Whelchel RL, Sanders TH, Thadhani NN (2014). Spall and dynamic yield behavior of an annealed aluminum–magnesium alloy. Scr. Mater..

[25] Deschamps J (2022). Additive laser excitation of giant nonlinear surface acoustic wave pulses. Phys. Rev. Appl..

[26] Hall CA (2000). Isentropic compression experiments on the Sandia Z accelerator. Phys. Plasmas.

[27] Cenna AA, Page NW, Kisi E, Jones MG (2011). Single particle impact tests using gas gun and analysis of high strain-rate impact events in ductile materials. Wear.

[28] Reiser A, Schuh CA (2023). Microparticle impact testing at high precision, higher temperatures, and with lithographically patterned projectiles. Small Methods.

[29] Bertin N, Carson R, Bulatov VV, Lind J, Nelms M (2023). Crystal plasticity model of BCC metals from large-scale MD simulations. Acta Mater..

[30] Wu CY, Li LY, Thornton C (2005). Energy dissipation during normal impact of elastic and elastic–plastic spheres. Int. J. Impact Eng..

[31] Wu CY, Li LY, Thornton C (2003). Rebound behaviour of spheres for plastic impacts. Int. J. Impact Eng..

[32] Sun Y, Veysset D, Nelson KA, Schuh CA (2020). The transition from rebound to bonding in high-velocity metallic microparticle impacts: jetting-associated power-law divergence. J. Appl. Mech..

[33] Hassani M, Veysset D, Nelson KA, Schuh CA (2020). Material hardness at strain rates beyond 10^6^ s^−1^ via high velocity microparticle impact indentation. Scr. Mater..

[34] Yu J, Wang G, Rong Y (2015). Experimental study on the surface integrity and chip formation in the micro cutting process. Procedia Manuf..

[35] Whitney, D. in *Comprehensive Hard Materials* Vol. 2 (eds Sarin, V. K., Llanes, L. & Mari, D.) 491–505 (Elsevier, 2014).

[36] Tabor D (1948). A simple theory of static and dynamic hardness. Proc. R. Soc. Lond. A. Math. Phys. Sci..

[37] Totten, G. E. & Mackenzie, D. S. in *ASM Handbook Vol. 4E, Heat Treating of Nonferrous Alloys 4E* (ed. Totten, G. E.) 325–334 (ASM International, 2016).

[38] Prime MB, Fensin SJ, Jones DR, Dyer JW, Martinez DT (2024). Multiscale Richtmyer-Meshkov instability experiments to isolate the strain rate dependence of strength. Phys. Rev. E.

[39] Akhondzadeh S, Kang M, Sills RB, Ramesh KT, Cai W (2023). Direct comparison between experiments and dislocation dynamics simulations of high rate deformation of single crystal copper. Acta Mater..

[40] Chen X, Xiong L, McDowell DL, Chen Y (2017). Effects of phonons on mobility of dislocations and dislocation arrays. Scr. Mater..

[41] Tanner AB, McGinty RD, McDowell DL (1999). Modeling temperature and strain rate history effects in OFHC Cu. Int. J. Plast..

[42] Mecking H, Kocks UF (1981). Kinetics of flow and strain-hardening. Acta Metall..

[43] Cai, W., Bulatov, V. V., Chang, J., Li, J. & Yip, S. in *Dislocations in Solids* Vol. 12 (eds Nabarro, F. R. N. & Hirth, J. P.) 1–80 (Elsevier, 2004).

[44] Mohamed FA, Langdon TG (1976). The determination of the activation energy for superplastic flow. Phys. Status Solidi.

[45] Lea LJ, Jardine AP (2018). Characterisation of high rate plasticity in the uniaxial deformation of high purity copper at elevated temperatures. Int. J. Plast..

[46] Lea L, Brown L, Jardine A (2020). Time limited self-organised criticality in the high rate deformation of face centred cubic metals. Commun. Mater..

[47] Katagiri K (2023). Transonic dislocation propagation in diamond. Science.

[48] Lee J-H, Loya PE, Lou J, Thomas EL (2014). Dynamic mechanical behavior of multilayer graphene via supersonic projectile penetration. Science.

[49] Tiamiyu AA, Sun Y, Nelson KA, Schuh CA (2021). Site-specific study of jetting, bonding, and local deformation during high-velocity metallic microparticle impact. Acta Mater..

[50] Lienhard J, Nelson KA, Schuh CA (2021). Melting and ejecta produced by high velocity microparticle impacts of steel on tin. J. Appl. Mech..

