# Supplementary material for: Metals strengthen with increasing temperature at extreme strain rates
Source: Nature. 2024 May 22;630(8015):91–5. doi: 10.1038/s41586-024-07420-1 (PMC11153132; doi:10.1038/s41586-024-07420-1)
Supplement: Supplementary file 1 — Supplementary Methods. [file 41586_2024_7420_MOESM1_ESM.docx]

Supplementary Materials for

**Metals strengthen with increasing temperature at extreme strain rates**

**Authors:** Ian Dowding^1^, Christopher A. Schuh^1,2*^

**Affiliations:**

^1^Department of Materials Science and Engineering, Massachusetts Institute of Technology; Cambridge, MA 02139, USA.

^2^Department of Materials Science and Engineering, Northwestern University; Evanston, IL 60208, USA.

*Corresponding author. Email: schuh@northwestern.edu

Supplementary Text

**S1. Dynamic strength and hardness**

The dynamic strength calculations follow the plasticity model by Wu et al. who simulated elastic-perfectly plastic spherical particle impacts ^1,2^. In their work, impacts across several orders of magnitude of impact velocities collapse to a single curve that is fit to a power-law relation, Eq. 1 in the main text. For impacts of an elastic impactor on an elastic perfectly plastic substrate *Y_d_* is the dynamic yield strength, *E*^*^ is the effective modulus of the impactor and substrate:

| $\frac{1}{E^{*}}=\frac{1-\upsilon_{1}^{2}}{E_{1}}+\frac{1-\upsilon_{2}^{2}}{E_{2}}$ | (S1) |
| --- | --- |

where *υ* and *E* are the Poisson ratio and Young’s moduli of each material, i.e., the impactor and substrate. The quantity *v*_y_ is the impact velocity when plastic deformation first initiates:

| $v_{y}=\left( \frac{26Y_{d}^{5}}{\rho E^{*4}} \right)^{\frac{1}{2}}$ | (S2) |
| --- | --- |

where *ρ* is the density of the substrate.

Although microballistic experiments are not elastic-perfectly plastic conditions, fitting with this model returns an average measure of *Y_d_* over a large range of impact velocities averaging strain- and rate-hardening, adiabatic heating, grain size evolution, etc., into an effective strength of the impact event.

***S1.1 Thermal strengthening in gold and titanium***

The thermal strengthening seen at extreme strain rates in Figs. 1-3 is not unique to copper. We have extended these results to two additional close packed metals for completeness. First, gold was selected because it is a face-centered cubic metal like copper, but is noble and therefore doesn’t have a native oxide layer and is thus likely chemically cleaner than copper. Second, titanium was selected to generalize the observation to a noncubic (hexagonal) crystal system.

Extended Data Figure 1(A) shows the impact velocity versus coefficient of restitution (CoR) curves for alumina impacts on pure gold at 20 °C (blue) and 97 °C (red). The solid blue and red lines show the same parabolic scaling that is seen in copper, and can therefore be used to extract the dynamic yield strength, *Y_d_*, by fitting with Eq. 1. A 77 °C increase in temperature led to ~5% increase in *Y_d_*; fitted values are shown inset to Extended Data Figure 1(A). Additionally, in Extended Data Figure 1(B), the dynamic hardness of titanium was measured for each impact at each temperature. In general, for impacts at the same strain rates, higher hardness values are measured for impacts at 97°C.

As with both copper and gold, the same trend is seen in titanium where a 180 °C increase in temperature led to a ~14% increase in *Y_d_*, Extended Data Figure 2(A). What is more, for impacts at the same strain rate hardness values at 200 °C were on average higher than hardness values at 20 °C, Extended Data Figure 2(B).

**S2. High strain rate strength model**

At high strain rates, the total strength of a pure metal can be broken down into its individual strengthening mechanisms: thermal, athermal, and drag strengthening.

***S2.1 Thermally activated dislocation motion***

The thermal strength component, σ_th_, of a metal arises from dislocation interactions with short-range barriers and, with sufficient thermal energy, can be overcome by thermal fluctuations alone. σ_th_ is a function of strain rate and temperature, and leads to conventional thermal softening of metals at all strain rates and temperatures ^3,4^.

| $\sigma_{th}=\left[ 1-\left( \frac{kT}{g_{0}\mu_{T}b^{3}}\ln\left( \frac{\dot{\varepsilon_{0}}}{\dot{\varepsilon}} \right) \right)^{\frac{1}{q}} \right]^{\frac{1}{p}}\sigma_{0}$ | (S3) |
| --- | --- |
| $\mu_{T}= \mu_{0}-\sqrt{a+\left( \frac{T}{T_{r}} \right)^{2}}$ | (S4) |

The thermal strength component is calculated with Eq. S3, where *σ_0_* is the stress to overcome the relevant short range barrier at 0 K, *k* is the Boltzmann’s constant, *T* is the temperature, *g_0_* is the activation energy normalized to 0 K, *μ_T_* is the temperature dependent shear modulus (Eq. S4), *b* is the magnitude of the Burgers vector, $\dot{\varepsilon_{0}}$ is a reference strain rate, $\dot{\varepsilon}$ is the strain rate, and *q* and *p* are fitting parameters based on the shape of the short-range barrier profile ^5^. For copper, the temperature dependent shear modulus, Eq. S4, $\mu_{0}$is the shear modulus at 0 K, 47.1 GPa, and *a* and *T_r_* are constants^3^ equal to 0.14 and 60.16, respectively.

***S2.2 Athermal strengthening***

Beyond dislocation interactions with short-range barriers, long-range barriers to dislocation motion (dislocation-dislocation interactions, dislocation-grain boundary interactions) also contribute to the total strength. These barriers however, cannot be overcome by thermal fluctuations alone and are therefore termed athermal strength contributions, *σ_a_* ^3^:

| $\sigma_{a}= \frac{\alpha_{G}\mu_{T}\sqrt{b}}{\sqrt{D}}+\alpha_{disl}\mu_{T}b\sqrt{\rho}$ | (S5) |
| --- | --- |

Dislocation-grain boundary strengthening effects can be captured in a Hall-Petch type relationship, the first term in Eq. S5. *D* is taken to be the final grain size after impact, on the order of several hundred nanometers based on copper microparticle impacts of Tiamiyu et. al ^6^, and *α_G_* is a dislocation-grain boundary interaction parameter, here 0.15. The second term in Eq. S5 relates dislocation-forest interactions to strength through the mobile dislocation density, *ρ* ( ~10^13^ m^-2^ at these strain rates in copper ^7^) and a dislocation-dislocation interaction parameter, *α_disl_*, equal to 0.5. Despite there being a small decrease in athermal strength with temperature due to a reduction in the temperature dependent shear modulus, these long-range obstacles cannot be overcome through increases in temperature.

***S2.3 Drag Strengthening***

Several sources of drag exist on moving dislocations in crystals, however under the temperature and strain rate conditions in this study, the dominant drag strengthening mechanisms are from interactions with phonons, namely phonon scattering and phonon viscosity.

| $\sigma_{d}= \frac{B_{d}}{\rho b^{2}}\dot{\varepsilon}$ | (S6) |
| --- | --- |
| $B_{d}=\frac{3kT}{5\cdot Ca^{3}}(1.8b)$ | (S7) |

Equations S6 and S7 are a standard model for the phonon drag strengthening mechanism ^8^, where *B_d_* is the damping constant. In copper at strain rates of 10^7^ and temperatures between 20 – 177 °C, *B_d_* is ~10^-5^ Pa∙sec, and is consistent with both shock wave loading ^7^ and atomistic simulations of copper at high rates ^9^. The damping constant can be calculated as the effective dislocation width, 1.8*b*, times the thermal energy density divided by five times the shear wave velocity, *C*, Eq. S7. The damping coefficient scales linearly with temperature and the lattice coefficient, *a*, marginally increases with temperature through the coefficient of thermal expansion. At a constant high rate, as in Fig. 3, when evaluating *σ_d_* as a function of temperature, the mobile dislocation density, *ρ*, is a constant ^7^, and the strength changes only through the change in temperature and lattice parameter. Increases in T dominate any increases in *a* giving rise to the increase in strength across all temperatures at high strain rates. At sufficiently low temperatures, below around ~100K, phonon drag effects become negligible. While other sources of drag strengthening are possible ^10^, such as electron drag ^11^, inertial effects ^12^, thermoelastic damping ^13^, and lattice friction ^14^, drag from phonon viscosity and phonon scattering is the dominant drag mechanism on mobile dislocations under these material and strain rate conditions.

**S3. Strain rate threshold for thermal hardening**

The temperature dependence of strength, thermal softening or thermal hardening, can be predicted through determination of the apparent activation energy for plasticity, *Q_app_*, Eq. 3 in the main text ^5,15^. Arrhenius type plots for fixed strain rates can be calculated based on Eq. S3, S5, and S6 with the local slopes used to calculate *Q_app_*. At a critical strain rate, *Q_app_* changes from positive to negative, indicating a change in plasticity from thermally activated to ballistic transport of dislocations. However, for determining when phonon drag effects become significant, *Q_app_* being negative is necessary but not alone sufficient for satisfying this condition. The derivative of the strain rate sensitivity parameter with stress at constant temperature, Eq. S8, must also be negative:

| $\left. \frac{{\partial\left( \left( \frac{\partial\ln\dot{\varepsilon}}{\partial\ln\sigma} \right) \right)}_{T}}{\partial\sigma} \right\vert_{T}<0$ | (S8) |
| --- | --- |

In the present case, when *Q_app_* crosses zero, the sensitivity parameter with stress is also negative, satisfying the phenomenological conditions for phonon drag to have a significant effect on strength. At any deformation rate beyond this threshold, thermal hardening dominates thermal softening due to phonon drag effects on moving dislocations.

**References for Supplementary Text**

1. Wu, C. Y., Li, L. Y. & Thornton, C. Energy dissipation during normal impact of elastic and elastic-plastic spheres. *Int. J. Impact Eng.* **32**, 593–604 (2005).

2. Wu, C. Y., Li, L. yuan & Thornton, C. Rebound behaviour of spheres for plastic impacts. *Int. J. Impact Eng.* **28**, 929–946 (2003).

3. Liu, R., Salahshoor, M., Melkote, S. N. & Marusich, T. A unified material model including dislocation drag and its application to simulation of orthogonal cutting of OFHC Copper. *J. Mater. Process. Technol.* **216**, 328–338 (2015).

4. Tanner, A. B., McGinty, R. D. & McDowell, D. L. Modeling temperature and strain rate history effects in OFHC Cu. *Int. J. Plast.* **15**, 575–603 (1999).

5. Kocks, U. F., Argon, A. S. & Ashby, M. F. Thermodynamics and Kinetics of Slip. *Progress in Materials Science Volume 19* 1–291 (1975).

6. Tiamiyu, A. A. *et al.* Nanotwinning-assisted dynamic recrystallization at high strains and strain rates. *Nat. Mater.* (2022). doi:10.1038/s41563-022-01250-0

7. Zaretsky, E. B. & Kanel, G. I. Response of copper to shock-wave loading at temperatures up to the melting point. *J. Appl. Phys.* **114**, (2013).

8. Wulf, G. L. High strain rate compression of titanium and some titanium alloys. *Int. J. Mech. Sci.* **21**, 713–718 (1979).

9. Kuksin, A. Y. & Yanilkin, A. V. Atomistic simulation of the motion of dislocations in metals under phonon drag conditions. *Phys. Solid State* **55**, 1010–1019 (2013).

10. Gurrutxaga–Lerma, B., Verschueren, J., Sutton, A. P. & Dini, D. The mechanics and physics of high-speed dislocations: a critical review. *Int. Mater. Rev.* **66**, 215–255 (2021).

11. Mason, W. P. Effect of Electron-Damped Dislocations on the Determination of Superconducting Energy Gaps of Metals. Phys. Rev. **143**, 229–235 (1966).

12. Eshelby, J. D. Uniformly moving dislocations. *Proc. Phys. Soc. Sect. A* **62**, 307–314 (1949).

13. Weiner, J. H. Thermoelastic dissipation due to high-speed dislocations. *J. Appl. Phys.* **29**, 1305–1307 (1958).

14. Hart, E. W. Lattice Resistance to Dislocation Motion at High Velocity. *Phys. Rev.* **98**, 1775–1776 (1955).

15. Mohamed, F. A. & Langdon, T. G. The determination of the activation energy for superplastic flow. *Phys. Status Solidi* **33**, 375–381 (1976).
